# Supplementary material for: Recurrent innovation of protein-protein interactions in the Drosophila piRNA pathway
Source: EMBO J. 2025 Apr 24;45(6):1909–32. doi: 10.1038/s44318-025-00439-8 (PMC12992792; doi:10.1038/s44318-025-00439-8)
Supplement: Supplementary file 2 — Appendix [file 44318_2025_439_MOESM2_ESM.pdf]

Appendix for

Recurrent innovation of protein-protein interactions in the Drosophila piRNA pathway

Sebastian Riedelbauch[1], Sarah Masser[2,5], Sandra Fasching[2], Sung-Ya Lin[3], Harpreet Kaur Salgania[4], Mie Aarup[1], Anja Ebert[1], Mandy Jeske[4], Mia Levine[3], Ulrich Stelzl[2,5,6], Peter Andersen[1,#]

1 Department of Molecular Biology and Genetics, Aarhus University, 8000 Aarhus C, Denmark.
2 Institute of Pharmaceutical Sciences, Pharmaceutical Chemistry, University of Graz, Austria.
3 Department of Biology, Epigenetics Institute, University of Pennsylvania, Philadelphia, PA, USA.
4 Heidelberg University Biochemistry Center (BZH), 69120 Heidelberg, Germany.
5 BioTechMed-Graz, Graz, Austria.
6 Field of Excellence BioHealth - University of Graz, Austria.
# Correspondence: pra@mbg.au.dk

Table of Contents

Page	Content	Description
1	Table of contents	
2	Appendix Figure S1	Positive selection test results of codeml analyses from PAML related to Figure 1B
3	Appendix Figure S2	Synteny analyses for CtBP, TfIIA–S, and UAP56 orthologs
4	Appendix Figure S3	Synteny analyses for rhi, del, and cuff orthologs
5	Appendix Figure S4	Appendix Figure S3. Synteny analyses for kipf, moon, and Trf2 orthologs
6	Appendix Figure S5	Synteny analyses for Nxf3 and Boot orthologs
7	Appendix Figure S6	Yeast-two-hybrid data evaluation and replication score calculation
8	Appendix Figure S7	Supplementary confocal imaging data related to Figure EV3F
9	Appendix Figure S8	Protein sequence alignment of CtBP from five Drosophila species
10	Appendix Table S1	Overview of the fly stock used in the study
11	Appendix Table S2	Overview DNA oligos used to clone the Y2H vectors

Gene	Species	range of % sequence analyzed	Test: M7 vs. M8 2ΔlogL (log ratio)	p value	Test: M8 vs. M8a 2ΔlogL (log ratio)	p value	positively selected sites (prob. > 95%))
Boot	*mel, sim sec, mau, ere, yak, tei, eug, bia, suz, tak, ele, rho, fic*	76 - 92	0.06	0.97	0.18	0.67	NA
CtBP	*mel, sim sec, mau, ere, yak, tei, eug, bia, suz, tak, ele, rho, fic*	77 - 97	0.00	1.00	0.00	1.00	NA
cuff	*mel, sim sec, mau, ere, yak, tei, eug, bia, suz, tak, ele, rho, fic*	92 - 96	1.28	0.53	0.36	0.36	NA
del	*mel, sim sec, mau, ere, yak, tei, eug, bia, suz, tak, ele, rho, fic*	55 - 71	9.85	0.01	0.77	0.38	NA
kipf	*mel, sim sec, mau, ere, yak, tei, eug, suz, tak, ele, fic*	50 -72	17.60	0.00	7.32	0.01	97P
moon	*mel, sim sec, mau, ere, yak, tei, eug, suz, tak, ele, rho, fic*	69 - 98	13.97	0.00	7.95	0.01	164E
Nxf3	*mel, sim sec, mau, ere, yak, tei, eug, bia, suz, tak, ele, rho, fic*	90 -94	1.67	0.43	0.86	0.35	NA
rhi	*mel, sim sec, mau, ere, yak, tei, eug, bia, suz, tak, ele, rho, fic*	14 - 93	12.26	0.00	7.48	0.01	57K, 70H, 74I
TFIIA-S	*mel, sim sec, mau, ere, yak, tei, eug, bia, suz, tak, ele, rho, fic*	99 - 100	0.00	1.00	0.00	1.00	NA
Trf2	*mel, sim sec, mau, ere, yak, tei, tak, rho*	50 - 70	95.03	0.00	69.49	0.00	***
Trf2-S	*mel, sim sec, mau, ere, yak, tei, eug, bia, suz, tak, ele, rho, fic*	77 - 90	2.87	0.24	0.98	0.32	NA
UAP56	*mel, sim sec, mau, ere, yak, tei, eug, bia, suz, tak, ele, rho, fic*	100	0.01	1.00	0.03	0.86	1.00

Appendix Figure S1. Positive selection test results of codeml analyses from PAML related to Figure 1B
Results of codeML analysis (PAML software package) of the genes involved in germline piRNA precursor biogenesis (Figure 1A) based on comparison of model M7 vs. M8 (more sensitive) or models M8 vs. M8a (stricter). We report positively selected sites inferred from the M8 model for genes that are significant for the M7 vs. M8 or M8a vs. M8 comparison. TRF2 residues under positive selection are marked with ***: 2G*, 4A**, 29F*, 54R*, 101T**, 102R*, 126N*, 157G**, 159S*, 180R*, 184S*, 200S*, 236F**, 291S*, 364F*, 367E*. Of note, all of these residues are part of the long unstructured C-terminal region of the long isoform of TRF2and not in the short isoform.

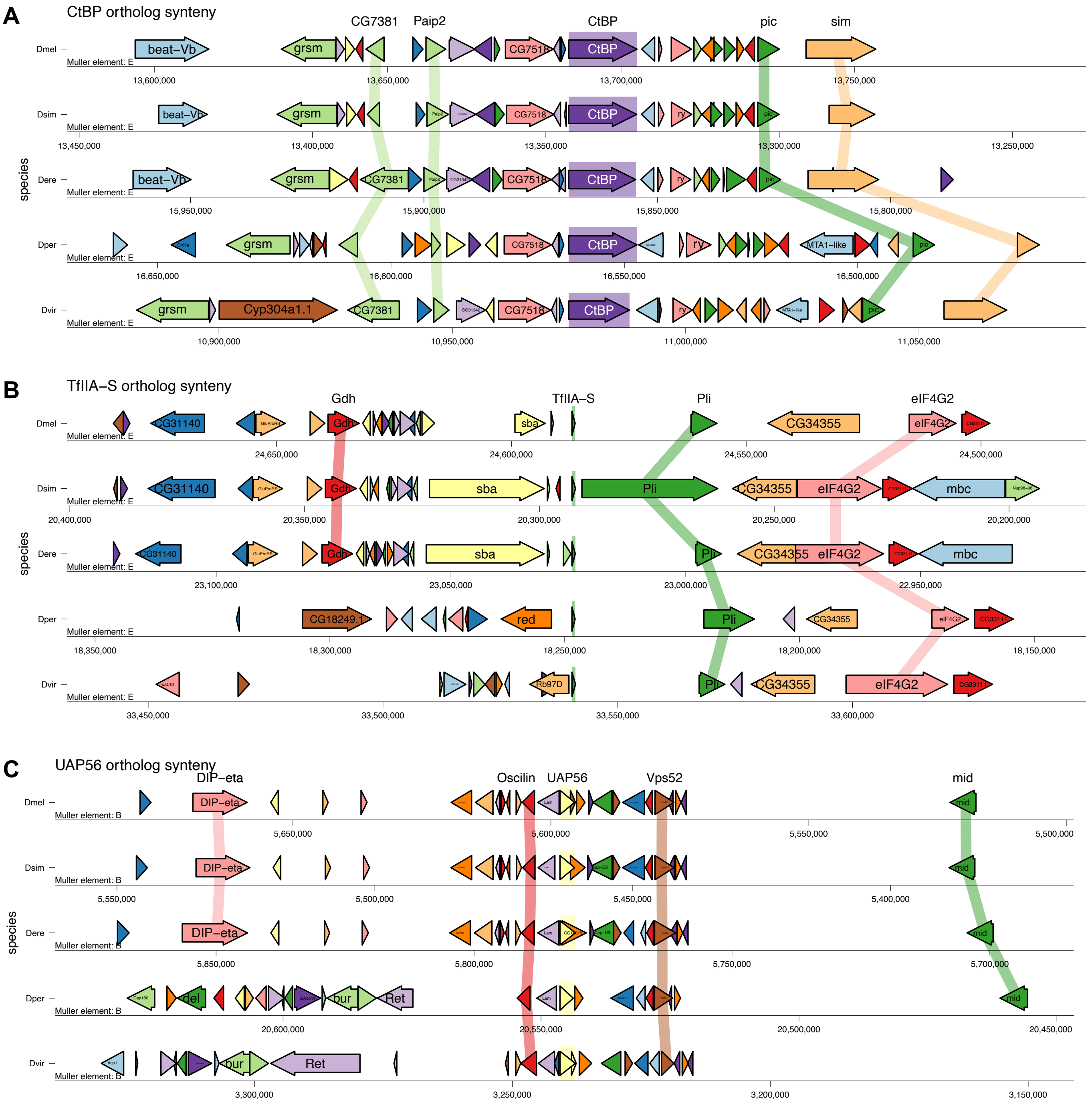

Appendix Figure S2. Synteny analyses for CtBP, TfIIA–S, and UAP56 orthologs
Gene annotations are shown in 100 kb-windows flanking either side of CtBP (A), TfIIA–S (B), and UAP56 (C). Genes are named by orthology to the *D. melanogaster* genes (see Methods for details). Selected genes present in multiple genomes are highlighted by vertical thick lines to indicate syntenic gene locations flanking the gene of interesting in the center. Genes are colored by orthology with color recycling so the same color can occur for different ortholog groups. The Muller Element chromosome segment is noted on the left side of the plot. Genome coordinates on the relevant Muller element assembly are noted below the gene annotations.

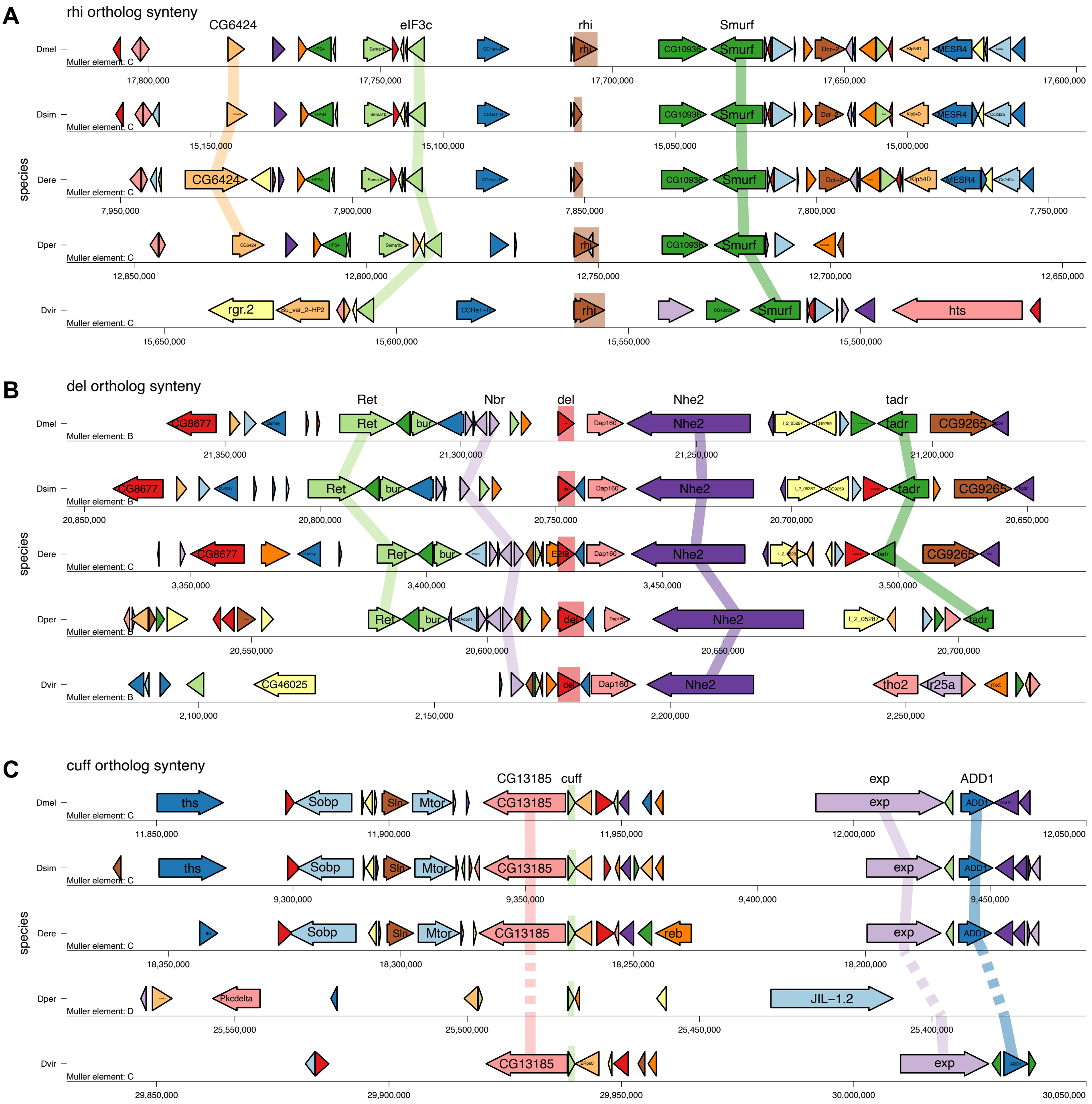

Appendix Figure S3. Synteny analyses for rhi, del, and cuff orthologs
Gene annotations are shown in 100 kb-windows flanking either side of rhi(A), del (B), and cuff (C). Genes are named by orthology to the *D. melanogaster* genes (see Methods for details). Selected genes present in multiple genomes are highlighted by vertical thick lines to indicate syntenic gene locations flanking the gene of interesting in the center. Genes are colored by orthology with color recycling so the same color can occur for different ortholog groups. The Muller Element chromosome segment is noted on the left side of the plot. Genome coordinates on the relevant Muller element assembly are noted below the gene annotations.

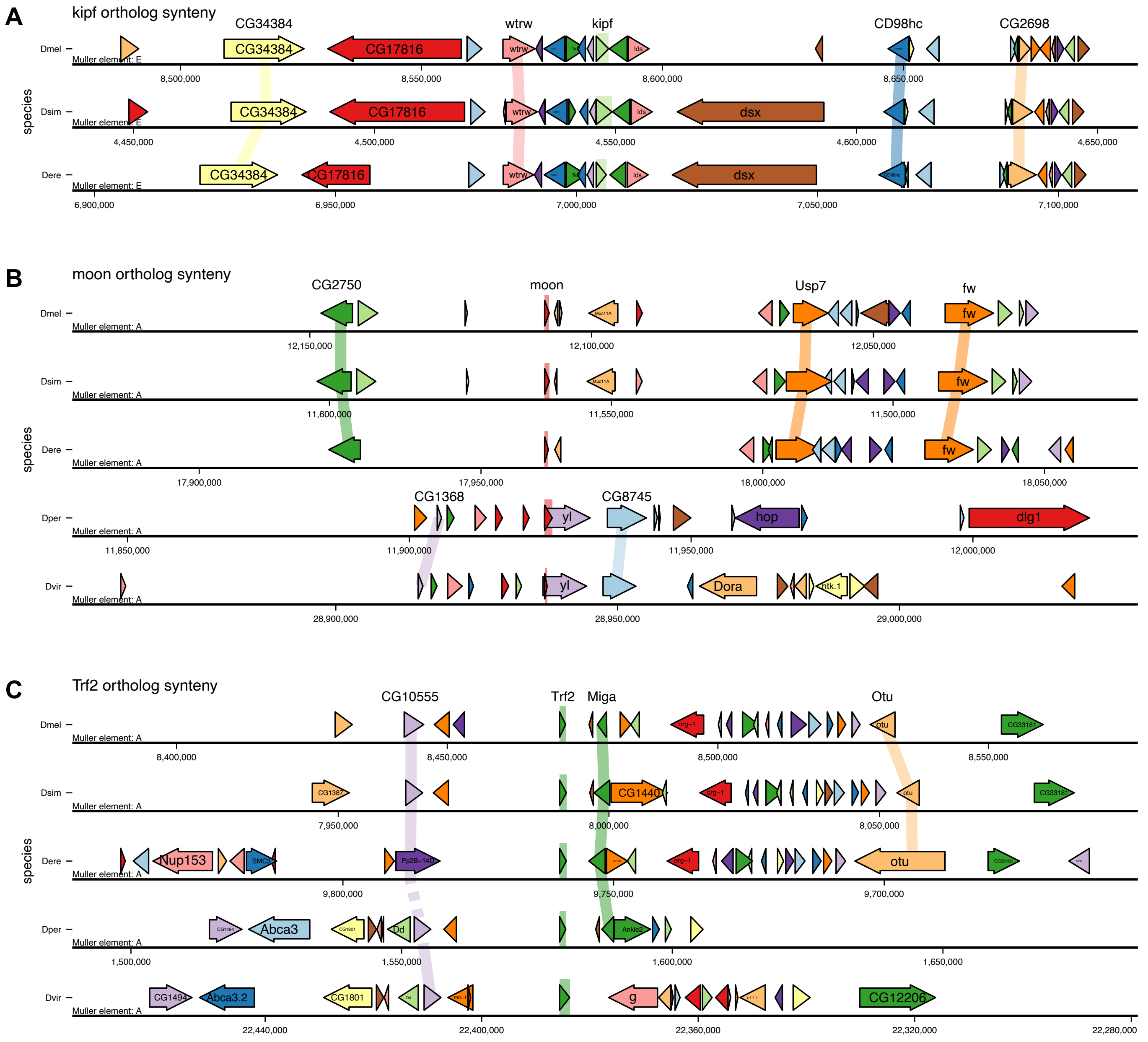

Appendix Figure S4. Synteny analyses for kipf, moon, and Trf2 orthologs
Gene annotations are shown in 100 kb-windows flanking either side of kipf (A), moon (B), and Trf2 (C). Genes are named by orthology to the *D. melanogaster* genes (see Methods for details). Selected genes present in multiple genomes are highlighted by vertical thick lines to indicate syntenic gene locations flanking the gene of interesting in the center. Genes are colored by orthology with color recycling so the same color can occur for different ortholog groups. The Muller Element chromosome segment is noted on the left side of the plot. Genome coordinates on the relevant Muller element assembly are noted below the gene annotations.

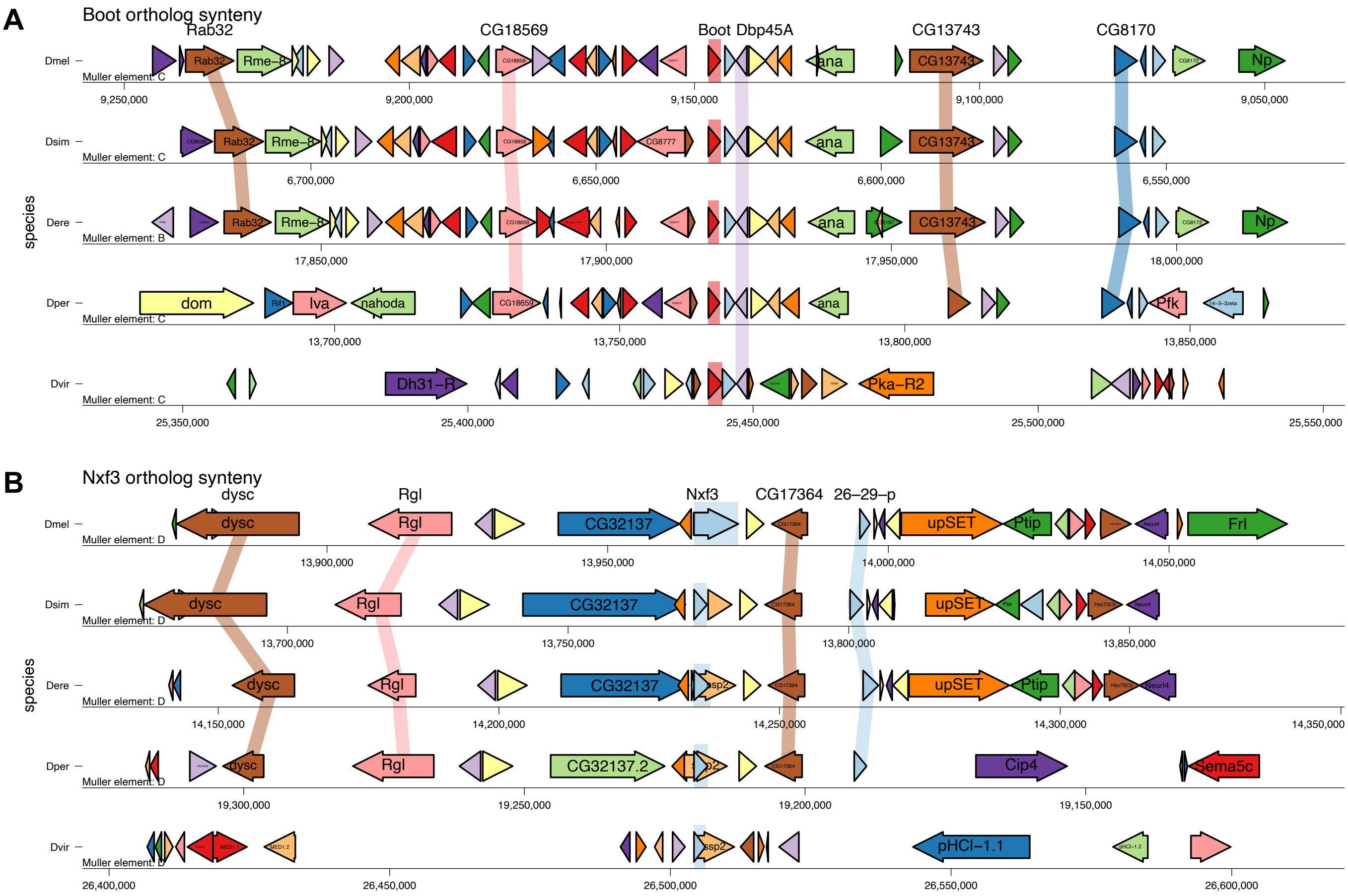

Appendix Figure S5. Synteny analyses for Nxf3 and Boot orthologs
Gene annotations are shown in 100 kb-windows flanking either side of Nxf3 (A) and Boot (B). Genes are named by orthology to the *D. melanogaster* genes (see Methods for details). Selected genes present in multiple genomes are highlighted by vertical thick lines to indicate syntenic gene locations flanking the gene of interesting in the center. Genes are colored by orthology with color recycling so the same color can occur for different ortholog groups. The Muller Element chromosome segment is noted on the left side of the plot. Genome coordinates on the relevant Muller element assembly are noted below the gene annotations.

A

①	Clone 11 genes from 5 species, transform and mate all-against-all	89,088	yeast matings
②	Drop autoreactive bait (10) and prey (2) and detect colony-forming matings	4,581	colonies
③	Aggregate colonies in technical and biological replicates	1,573	interactions detected
④	Drop weakly replicated interactions with weak growth	627	high-conf. interactions
⑤	Collapse all vectors supporting each tested protein-protein interaction (PPIs)	263	PPIs

B **Interaction replication score calculation:**

1. Per vector pair score: colonies [Count] / Interactions tested (max 1)
2. If reproduced in biological replicates: Use the highest individual score
3. Add the highest scores of each vector combination (max 8)

Final score:	< 0.5	≥ 0.5	≥ 0.75	≥ 1	≥ 2	≥ 4
# of interactions:	263	238	199	172	92	46
→ intra-species:	64	55	46	42	29	14
→ inter-species:	199	183	153	130	63	32

Appendix Figure S6. Yeast-two-hybrid data evaluation and replication score calculation
(A) Table showing the summary statistics following each step in the yeast-to-hybrid screen data evaluation workflow, resulting in the detection of 263 individual protein-protein interactions. (B) Overview of the calculation of the yeast-two-hybrid growth replication score, including summary statistics for six different score thresholds. Based on congruence with previous data describing protein-protein interactions within the tested network a threshold of ≥ 0.75 was selected for calling positive interactions in our screen.

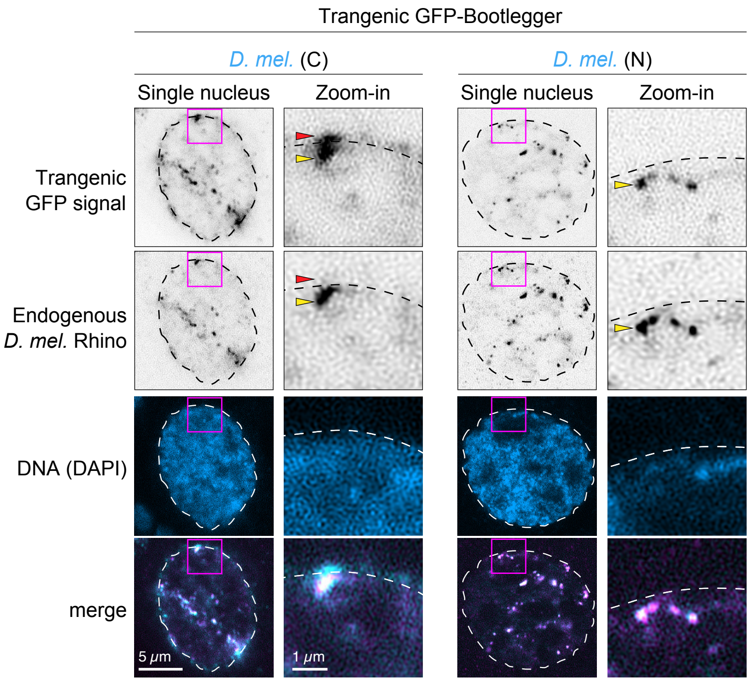

Appendix Figure S7. Supplementary confocal imaging data related to Figure EV3F
Confocal microscopy images showing the localization of endogenous *D. melanogaster* Rhino (anti-Rhi IF) and GFP-tagged transgenic D. melanogaster Bootlegger tagged at the indicated terminus. The single nucleus images are directly reused from Figure EV3F, while the zoom-in images are enlarged versions of the region outlined by magenta boxes of the same images. Dashed line: nuclear border as determined by DAPI staining. Yellow arrow heads highlight co-localizing foci of endogenous Rhino IF signal and transgenic GFP-tagged proteins. Red arrow heads indicate cytoplasmic peri-nuclear foci of GFP-Bootlegger adjacent to nuclear foci (absent for N-terminally tagged Bootlegger).

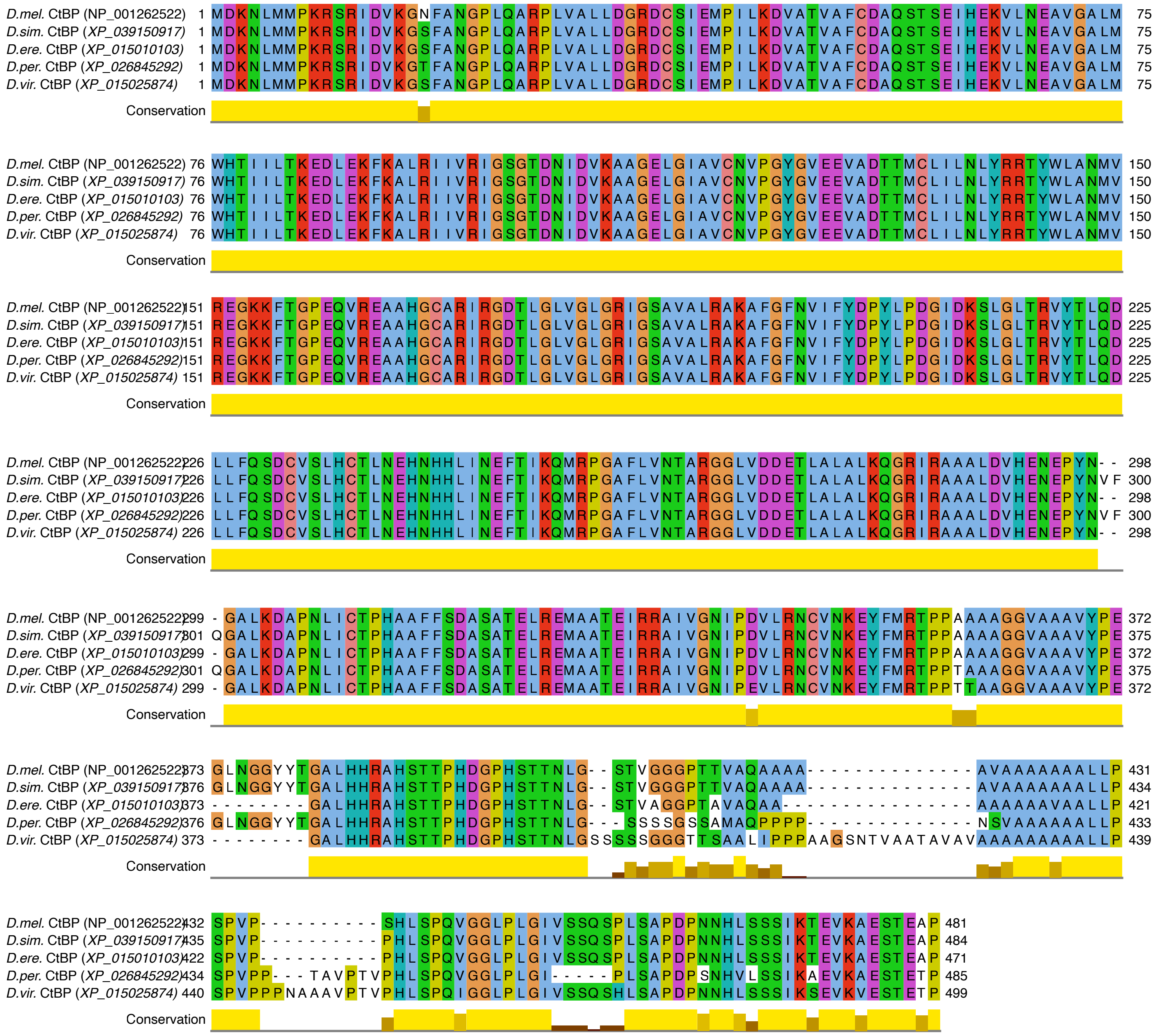

Appendix Figure S8. Protein sequence alignment of CtBP from five Drosophila species

The protein sequences were retrieved from NCBI using the noted accession numbers, aligned using the MUSCLE program (Edgar 2004) and displayed using Jalview. Amino acids are colored according to the 'Clustal' color scheme. Conservation is shown as bar diagrams with maximum values of 10, representing full conservation in all included species.

Fly stock	Genotype	Description	Source
FS21	*w;;CG13741_GFP/Precission/V5/3xFLAG [attP2]/TM3, Sb;*	*Dm*Bootlegger-GFP	(ElMaghraby et al. 2019)
FS52	*w;;3xFLAG/V5/Precission/GFP(replacing CG13741 CDS) + NLS [attP2]/TM3, Sb;*	GFP in ovaries	(ElMaghraby et al. 2019)
FS368	w^{1118}*;pRASA>NLAP-DmDeadlock*$^{w+}$ *[attP40];*	GFP-*Dm*Deadlock	This study
FS369	w^{1118}*;pRASA>NLAP-DsDeadlock*$^{w+}$ *[attP40]/CyO;*	GFP-*Ds*Deadlock	This study
FS370	w^{1118}*;pRASA>NLAP-DeDeadlock*$^{w+}$ *[attP40];*	GFP-*De*Deadlock	This study
FS371	w^{1118}*;pRASA>NLAP-DvDeadlock*$^{w+}$ *[attP40];*	GFP-*Dv*Deadlock	This study
FS372	w^{1118}*;pRASA>NLAP-DmBootlegger*$^{w+}$ *[attP40];*	GFP-*Dm*Bootlegger	This study
FS373	w^{1118}*;pRASA>NLAP-DsBootlegger*$^{w+}$ *[attP40];*	GFP-*Ds*Bootlegger	This study
FS374	w^{1118}*;pRASA>NLAP-DmRhino*$^{w+}$ *[attP40];*	GFP-*Dm*Rhino	This study
FS375	w^{1118}*;pRASA>NLAP-DpRhino*$^{w+}$ *[attP40];*	GFP-*Dp*Rhino	This study
FS376	w^{1118}*;pRASA>DmKipferl-CLAP*$^{w+}$ *[attP40];*	*Dm*Kipferl-GFP	This study
FS377	w^{1118}*;pRASA>-DeKipferl-CLAP*$^{w+}$ *[attP40];*	*De*Kipferl-GFP	This study

Appendix Table S1. Overview of the fly stock used in the study
See Methods section for details

Name	Sequence
SR295_BP_Dm_Moon_fw	GGGGACAAGTTTGTACAAAAAAGCAGGCTCCACCATGGCCAAGATATTGCCCACC
SR296_BP_Dm_Moon_rv	GGGGACCACTTTGTACAAGAAAGCTGGGTCCATTTCCTGGCCTGCGACC
SR297_BP_Ds_Moon_fw	GGGGACAAGTTTGTACAAAAAAGCAGGCTCCACCATGGCCAAGATTTTGCCCAC
SR298_BP_Ds_Moon_rv	GGGGACCACTTTGTACAAGAAAGCTGGGTCCGGGCGACCAATGTTGTC
SR299_BP_De_Moon_fw	GGGGACAAGTTTGTACAAAAAAGCAGGCTCCACCATGATTGGTGACAGTGGCAACT
SR300_BP_De_Moon_rv	GGGGACCACTTTGTACAAGAAAGCTGGGTCGTCTAGCTTGTCCGAGGTAACC
SR301_BP_Dv_Moon_fw	GGGGACAAGTTTGTACAAAAAAGCAGGCTCCACCATGATTGAACTAAGCGACACTGAAAAAAG
SR302_BP_Dv_Moon_rv	GGGGACCACTTTGTACAAGAAAGCTGGGTCATCAACTTGCATCTGCAGCAAT
SR303_BP_Dm_TfIIA-S_fw	GGGGACAAGTTTGTACAAAAAAGCAGGCTCCACCATGTCGTATCAACTGTACC
SR304_BP_Dm_TfIIA-S_rv	GGGGACCACTTTGTACAAGAAAGCTGGGTCGAACTCGCCGCTCTTGC
SR305_BP_Ds_TfIIA-S_fw	GGGGACAAGTTTGTACAAAAAAGCAGGCTCCACCATGTCGTATCAACTGTACC
SR306_BP_Ds_TfIIA-S_rv	GGGGACCACTTTGTACAAGAAAGCTGGGTCGAACTCGCCGCTCTTGC
SR307_BP_De_TfIIA-S_fw	GGGGACAAGTTTGTACAAAAAAGCAGGCTCCACCATGTCGTATCAACTGTACC
SR308_BP_De_TfIIA-S_rv	GGGGACCACTTTGTACAAGAAAGCTGGGTCGAACTCGCCGCTCTTGCC
SR309_BP_Dv_TfIIA-S_fw	GGGGACAAGTTTGTACAAAAAAGCAGGCTCCACCATGTCTTATCAATTATATCGCAACAC
SR310_BP_Dv_TfIIA-S_rv	GGGGACCACTTTGTACAAGAAAGCTGGGTCATTGAAATCGCCGCTTTTGCC
SR311_BP_Dmse_Boot_fw	GGGGACAAGTTTGTACAAAAAAGCAGGCTCCACCATGACGGATTTCAATGC
SR312_BP_Dm_Boot_rv	GGGGACCACTTTGTACAAGAAAGCTGGGTCATTAAAACGTTGCAGTTGGTCCC
SR313_BP_Ds_Boot_rv	GGGGACCACTTTGTACAAGAAAGCTGGGTCGTTAAAACGATGCAGTTCGTCCC
SR314_BP_De_Boot_rv	GGGGACCACTTTGTACAAGAAAGCTGGGTCGTTAAAACGCTGCTGTTCGTC
SR315_BP_Dv_Boot_fw	GGGGACAAGTTTGTACAAAAAAGCAGGCTCCACCATGACTTCGTTTAATGCG
SR316_BP_Dv_Boot_rv	GGGGACCACTTTGTACAAGAAAGCTGGGTCCTTAAAGCGATGATACATTCGCAGC
SR317_BP_Dm_Nxf3_fw	GGGGACAAGTTTGTACAAAAAAGCAGGCTCCACCATGCAAATATTTGCCACTTTCAAATCCCACGAGCCGGACAGCCGCTTGATCCAAAACTTCAAAAAGCACGTGAATTCGCAGTC
SR318_BP_Dm_Nxf3_rv	GGGGACCACTTTGTACAAGAAAGCTGGGTCAAGGTCATCGCATCCAAACAGG
SR319_BP_Ds_Nxf3_fw	GGGGACAAGTTTGTACAAAAAAGCAGGCTCCACCATGCGAATCTTTGACAATTCCAAATCCCACGAGCCG
SR320_BP_Ds_Nxf3_rv	GGGGACCACTTTGTACAAGAAAGCTGGGTCAAGGTCATTGCATCCGAACATTAGG
SR321_BP_De_Nxf3_fw	GGGGACAAGTTTGTACAAAAAAGCAGGCTCCACCATGCGAATCTTGGTCCAAAATTCCAAATCCCACG
SR322_BP_De_Nxf3_rv	GGGGACCACTTTGTACAAGAAAGCTGGGTCAAAATCATCACATCCAATCAGGACGTC
SR323_BP_Dv_Nxf3_fw	GGGGACAAGTTTGTACAAAAAAGCAGGCTCCACCATGAGTGTTTTCCAAAAGTTAGAGGAAAACGGCCTTAGAAATGG
SR324_BP_Dv_Nxf3_rv	GGGGACCACTTTGTACAAGAAAGCTGGGTCAAGCACATCGTCACTGATCACCA
SR325_BP_Dm_Cuff_fw	GGGGACAAGTTTGTACAAAAAAGCAGGCTCCACCATGAATTCTAATTACACAATATTAAACATCCGGG
SR326_BP_Dm_Cuff_rv	GGGGACCACTTTGTACAAGAAAGCTGGGTCAACTATAGAAGACATGGTTTGCAAATTCG
SR327_BP_Ds_Cuff_fw	GGGGACAAGTTTGTACAAAAAAGCAGGCTCCACCATGAATTCTAATTATAAAATATTGAACATCCAGGCTC
SR328_BP_Ds_Cuff_rv	GGGGACCACTTTGTACAAGAAAGCTGGGTCTTGTTTTTGGTAAACTGTGGAAGACATG
SR329_BP_De_Cuff_fw	GGGGACAAGTTTGTACAAAAAAGCAGGCTCCACCATGATATCTAATTACAAAATATTGAACATCCAGGC
SR330_BP_De_Cuff_rv	GGGGACCACTTTGTACAAGAAAGCTGGGTCAACTCTGGTAGACTTGGCTTGC
SR331_BP_Dv_Cuff_fw	GGGGACAAGTTTGTACAAAAAAGCAGGCTCCACCATGTATTCAAATTATAAAATTTTAAATCTTAATGCACGCTC
SR332_BP_Dv_Cuff_rv	GGGGACCACTTTGTACAAGAAAGCTGGGTCAATTTGATGTTGAACTTGTGCATTCAG
SR333_De_Nxf3_lo_fw	GGTCCAAAATTCCAAATCCCACGAGCCGGGCAGCCGCCTGGGCGTGAATCTCAAGGCGCACGTGAATTCACAGTCGCAGCTTGCCGATCTGCGAAAAAATCTGAATGAAAAAAGTCAAATTCGAGAATACGTACAAAAGCTAATTGGAAATGAACAGCATATGGGATCCGTGCTGGAC
SR334_Ds_Nxf3_lo_fw	ATTCCAAATCCCACGAGCCGGGCAGCCGCCTGGGCGTAAACCTCAAAAAGCACGTGAATTCACAGTCGCCGCTTGC
SR335_BP_Dm_Rhino_fw	GGGGACAAGTTTGTACAAAAAAGCAGGCTCCACCATGTCTCGCAACCATCAGCG
SR336_BP_Dm_Rhino_rv	GGGGACCACTTTGTACAAGAAAGCTGGGTCCTTGGGCACAATGATCCTCAAG
SR337_BP_Ds_Rhino_fw	GGGGACAAGTTTGTACAAAAAAGCAGGCTCCACCATGTCTCGCAAAAATCAACGACC
SR338_BP_Ds_Rhino_rv	GGGGACCACTTTGTACAAGAAAGCTGGGTCCTTGAGCACAGTGGTCCTC
SR339_BP_De_Rhino_fw	GGGGACAAGTTTGTACAAAAAAGCAGGCTCCACCATGTCTGGCGAAGCTCAACG
SR340_BP_De_Rhino_rv	GGGGACCACTTTGTACAAGAAAGCTGGGTCCTTGTGCACAGCGATCTTCAAG
SR341_BP_Dv_Rhino_fw	GGGGACAAGTTTGTACAAAAAAGCAGGCTCCACCATGTCCGAGGGCAATCGTC
SR342_BP_Dv_Rhino_rv	GGGGACCACTTTGTACAAGAAAGCTGGGTCATCTAGCGGACTCCGTAGCTG
SR343_BP_Dms_UAP56_fw	GGGGACAAGTTTGTACAAAAAAGCAGGCTCCACCATGGCCGACAATGACGATCTT
SR344_BP_Dms_UAP56_rv	GGGGACCACTTTGTACAAGAAAGCTGGGTCGCGTCCCTCAATGTATGTAGAGAG
SR345_BP_Dv_UAP56_rv	GGGGACCACTTTGTACAAGAAAGCTGGGTCGCGTCCCTCAATGTATGTGGAC
SR346_BP_Dm_Kipf_fw	GGGGACAAGTTTGTACAAAAAAGCAGGCTCCACCATGATGCACAGCGCGAAGAAC
SR347_BP_Dm_Kipf_rv	GGGGACCACTTTGTACAAGAAAGCTGGGTCCATCGCTATTACGTTGTTGCTGTG
SR348_BP_Ds_Kipf_fw	GGGGACAAGTTTGTACAAAAAAGCAGGCTCCACCATGATAAAGAGCGCGAAGAACGT
SR349_BP_Ds_Kipf_rv	GGGGACCACTTTGTACAAGAAAGCTGGGTCATTCAATGGCTGCTTTTCAATATTAAAACC
SR350_BP_De_Kipf_fw	GGGGACAAGTTTGTACAAAAAAGCAGGCTCCACCATGAAGAGCGCGAAGAACGC
SR351_BP_De_Kipf_rv	GGGGACCACTTTGTACAAGAAAGCTGGGTCATGTGCTGCTTTCAATGGCTG
SR352_BP_Ds_HP1D2_fw	GGGGACAAGTTTGTACAAAAAAGCAGGCTCCACCATGCGCAACCCACAGC
SR353_BP_Ds_HP1D2_rv	GGGGACCACTTTGTACAAGAAAGCTGGGTCGGCATCTTTGTAATTTGGGAAGGC
SR354_BP_Dm_Del_fw	GGGGACAAGTTTGTACAAAAAAGCAGGCTCCACCATGGAAAAGTTGGACAAAATAAGGATGAG
SR355_BP_Dm_Del_rv	GGGGACCACTTTGTACAAGAAAGCTGGGTCATCAAAATTATGTATATTGATCGCATATTCATTGG
SR356_BP_Ds_Del_fw	GGGGACAAGTTTGTACAAAAAAGCAGGCTCCACCATGGAAAACTTGGCTAAAATAAGGATGAG
SR357_BP_Ds_Del_rv	GGGGACCACTTTGTACAAGAAAGCTGGGTCATCAAAATGATGTATATTGGTCGTATATTCATTGG
SR358_BP_De_Del_fw	GGGGACAAGTTTGTACAAAAAAGCAGGCTCCACCATGGTCACACTGGAAAGAACCG
SR359_BP_De_Del_rv	GGGGACCACTTTGTACAAGAAAGCTGGGTCATCTAGGTAGTTTTTATTGTTCGCATATTCATTTG
SR360_BP_Dv_Del_fw	GGGGACAAGTTTGTACAAAAAAGCAGGCTCCACCATGCGTCGCCGTGATATTTTAC
SR361_BP_Dv_Del_rv	GGGGACCACTTTGTACAAGAAAGCTGGGTCAAAATCGCTCAAGTCGATGTTATCC
SR362_BP_Dm_Trf2_fw	GGGGACAAGTTTGTACAAAAAAGCAGGCTCCACCATGAATGAGCACGAG
SR363_BP_Dm_Trf2_rv	GGGGACCACTTTGTACAAGAAAGCTGGGTCGTAACGTGGACGCTTATTCTGC
SR364_BP_Ds_Trf2_fw	GGGGACAAGTTTGTACAAAAAAGCAGGCTCCACCATGATCACGGCCAA
SR365_BP_Ds_Trf2_rv	GGGGACCACTTTGTACAAGAAAGCTGGGTCGTAACGCGGACGCTTATTCT
SR366_BP_De_Trf2_fw	GGGGACAAGTTTGTACAAAAAAGCAGGCTCCACCATGACTGTGATTGATGACG
SR367_BP_De_Trf2_rv	GGGGACCACTTTGTACAAGAAAGCTGGGTCGTAACGTGGACGCTTGTTC
SR368_BP_Dv_Trf2_fw	GGGGACAAGTTTGTACAAAAAAGCAGGCTCCACCATGGCCGAGCC
SR369_BP_Dv_Trf2_rv	GGGGACCACTTTGTACAAGAAAGCTGGGTCGTAGCGCGGACGTTTG
SR370_BP_Dm_CtBP_fw	GGGGACAAGTTTGTACAAAAAAGCAGGCTCCACCATGGACAAAAATCTGATGATGCCGAAG
SR371_BP_all_CtBP_rv	GGGGACCACTTTGTACAAGAAAGCTGGGTCCGGCGCCTCCGTTGAC
SR372_BP_Ds_CtBP_rv	GGGGACCACTTTGTACAAGAAAGCTGGGTCCGGCGCCTCCGTTG
SR373_BP_De_CtBP_rv	GGGGACCACTTTGTACAAGAAAGCTGGGTCACGGCGCCTCCGTT
SR374_BP_Dv_CtBP_rv	GGGGACCACTTTGTACAAGAAAGCTGGGTCCGGTGTTTCCGTTGACTCG

Appendix Table S2. Overview DNA oligos used to clone the Y2H vectors
See Methods section for details
