# Supplementary material for: Recurrent innovation of protein-protein interactions in the Drosophila piRNA pathway
Source: EMBO J. 2025 Apr 24;45(6):1909–32. doi: 10.1038/s44318-025-00439-8 (PMC12992792; doi:10.1038/s44318-025-00439-8)
Supplement: Supplementary file 6 — Source data Fig. 2 [file 44318_2025_439_MOESM6_ESM.zip › SD_Figure_2/scripts/piEVO_Y2H_matrix_analysis.html]

Plotting of piRNA pathway Y2H matrix screen data


# Plotting of piRNA pathway Y2H matrix screen data

#### 2025-02-18

# Overview

- Aim: to visualize Y2H data investigating piRNA pathway protein
  interactions across Drosophila evolution
- Data: Y2H matrix screen scores

# 1. Set up working environment

### Load packages

```
library(tidyverse) # compilation of tidy packages
```

```
## Warning: package 'lubridate' was built under R version 4.3.3
```

```
library(RColorBrewer)
library(plotly) # for interactive plots
library(ggh4x) # for nested facets in ggplot2
```

```
## Warning: package 'ggh4x' was built under R version 4.3.3
```

```
library(cowplot) # to fix plot sizes in saved files
```

### Set up folder paths

```
getCurrentFileLocation <-  function()
{
    this_file <- commandArgs() %>% 
    tibble::enframe(name = NULL) %>%
    tidyr::separate(col=value, into=c("key", "value"), sep="=", fill='right') %>%
    dplyr::filter(key == "--file") %>%
    dplyr::pull(value)
    if (length(this_file)==0)
    {
      this_file <- rstudioapi::getSourceEditorContext()$path
    }
    return(dirname(this_file))
} # source: https://stackoverflow.com/questions/47044068/get-the-path-of-current-script

# Define and create folders:
workDIR = gsub("scripts", "", getCurrentFileLocation())
dataDIR = paste(workDIR, "Y2H_data/", sep = "")
outputDIR <- paste(workDIR, "output/", sep="")
# Create the output directory. Will ignore if it already exists
dir.create(file.path(outputDIR), showWarnings = FALSE, recursive = TRUE)


# Set color palette:
mycolors <- c("white", brewer.pal(n = 9, "YlOrRd")[3:7])

# Save ggplot2 aestetics theme to variable
line_width_1pt <- 1 / (72.27 / 25.4) / (72.27 / 96) # explanation: https://github.com/tidyverse/ggplot2/issues/3358

mytheme <- theme(panel.background=element_blank(),
                 # panel.grid = element_blank(),
                 axis.ticks = element_line(colour = "black", linewidth = line_width_1pt/4),
                 strip.text = element_text(size = 6, color="black"),
                 axis.text = element_text(size = 7, color="black"),
                 axis.title = element_text(size = 7), 
                 plot.title = element_text(size = 8),
                 plot.subtitle = element_text(size = 7),
                 text=element_text(family="Helvetica"),
                 plot.background = element_rect(fill='transparent', color=NA), #transparent plot bg
                 # panel.grid.major = element_blank(), #remove major gridlines
                 # panel.grid.minor = element_blank(), #remove minor gridlines
                 legend.background = element_rect(fill='transparent'), #transparent legend bg
                 legend.box.background = element_rect(fill='transparent'), #transparent legend panel
                 legend.text = element_text(size=7),
                 plot.margin = margin(t = 5, r = 5, b = 5, l = 5, unit = "pt"), 
                 legend.position = "right", legend.justification="right", legend.box.spacing = unit(40, "pt")
)
```

# 2. Read in and organize data

```
### Read in and format Y2H score data:
Y2H_file <- file.path(dataDIR, "Riedelbauh_Y2H_PPIs_cutoff_0_25.tsv")

species_order <- c("D mel", "D sim", "D ere", "D per", "D vir")

Y2H_data <- read_tsv(Y2H_file, show_col_types = FALSE) %>%
  mutate(Species_1 = recode(Species_1,
                            "Dm" = "D mel",
                            "Ds" = "D sim",
                            "De" = "D ere",
                            "Dp" = "D per",
                            "Dv" = "D vir"),
         Species_2 = recode(Species_2,
                            "Dm" = "D mel",
                            "Ds" = "D sim",
                            "De" = "D ere",
                            "Dp" = "D per",
                            "Dv" = "D vir")) %>%
  mutate(
    Protein_1 = factor(Protein_1, levels = rev(unique(Protein_1))),
    Protein_2 = factor(Protein_2, levels = unique(Protein_1)),
    Species_1 = factor(Species_1, levels = rev(species_order)),
    Species_2 = factor(Species_2, levels = species_order)
  )
```

# 3. Plot the Y2H replication score data

```
# Calculate grid line positions from protein ortholog (species) numbers
grid_positions <- Y2H_data %>%
  group_by(Protein_1) %>%
  summarise(unique_species_count = n_distinct(Species_1)) %>%
  pull(unique_species_count)

# Define a round function that is not round-to-even (the data is not continous):
true_round <- function(number, digits) {
  posneg <- sign(number)
  number <- abs(number) * 10^digits
  number <- number + 0.5 + sqrt(.Machine$double.eps)
  number <- trunc(number)
  number <- number / 10 ^ digits
  number * posneg
}

# Make plot from Figure EV8, Riedelbauch et al. 2025:
p <- ggplot(Y2H_data, aes(x = interaction(Species_2, Protein_2),
                          y = interaction(Species_1, Protein_1), fill = Value,
                          text = paste(
                            Protein_1, " (", Species_1, ") vs ",
                            Protein_2, " (", Species_2, ")",
                            "\nY2H repl. score: ", Value,
                            "\nCategory: ", PPI_cat,
                            sep = ""))) +
  geom_tile(color = "grey", lwd = line_width_1pt/4, linetype = 1) +
  geom_text(data = Y2H_data %>% filter(Value > 0),
            aes(label = true_round(Value, 1)), color = "black", size = 2) +
  scale_fill_gradientn(colours = mycolors) + # gradient as displayed in EV8
  # scale_fill_gradient(low = "white", high = "red") + # alternative two-color gradient
  geom_hline(yintercept = cumsum(grid_positions) + 0.5, lwd = line_width_1pt/4, linetype = 1) +
  geom_vline(xintercept = cumsum(rev(grid_positions)) + 0.5, lwd = line_width_1pt/4, linetype = 1) +
  mytheme +
  # theme_minimal() +
  labs(title = NULL, x = NULL, y = NULL, fill = "Value") +
  theme(axis.text.x = element_text(angle = 90, hjust = 0)) +
  scale_x_discrete(position = "top", guide = "axis_nested") +
  scale_y_discrete(guide = "axis_nested") +
  # coord_fixed() +
  theme(axis.ticks = element_line(colour = "black", linewidth = line_width_1pt/4))

ggplotly(p, tooltip = c("text"))
```

```
# Format for saving as pdf
legend <- get_plot_component(p, 'guide-box-right', return_all = TRUE)
```

```
## Warning: `guide_axis_nested()` was deprecated in ggh4x 0.3.0.
## ℹ Please use `legendry::guide_axis_nested()` instead.
## ℹ The deprecated feature was likely used in the ggplot2 package.
##   Please report the issue at <https://github.com/tidyverse/ggplot2/issues>.
## This warning is displayed once every 8 hours.
## Call `lifecycle::last_lifecycle_warnings()` to see where this warning was
## generated.
```

```
## Warning: The S3 guide system was deprecated in ggplot2 3.5.0.
## ℹ It has been replaced by a ggproto system that can be extended.
## This warning is displayed once every 8 hours.
## Call `lifecycle::last_lifecycle_warnings()` to see where this warning was
## generated.
```

```
p <- p + theme(legend.position = "none")
final_plot <- plot_grid(p, legend, ncol = 2, rel_widths = c(1, 0.2))

# Save the produced plot as .pdf file:
fileVAR = paste(outputDIR, "Riedelbauch_2025_EV2_Y2Hmatrix_plot.pdf", sep="")
ggsave(filename = fileVAR, plot=final_plot, width=22, height=15, units="cm", useDingbats=FALSE, bg='transparent')
```
