# Supplementary material for: Recurrent innovation of protein-protein interactions in the Drosophila piRNA pathway
Source: EMBO J. 2025 Apr 24;45(6):1909–32. doi: 10.1038/s44318-025-00439-8 (PMC12992792; doi:10.1038/s44318-025-00439-8)
Supplement: Supplementary file 10 — Expanded View Figures [file 44318_2025_439_MOESM10_ESM.pdf]

Expanded View Figures

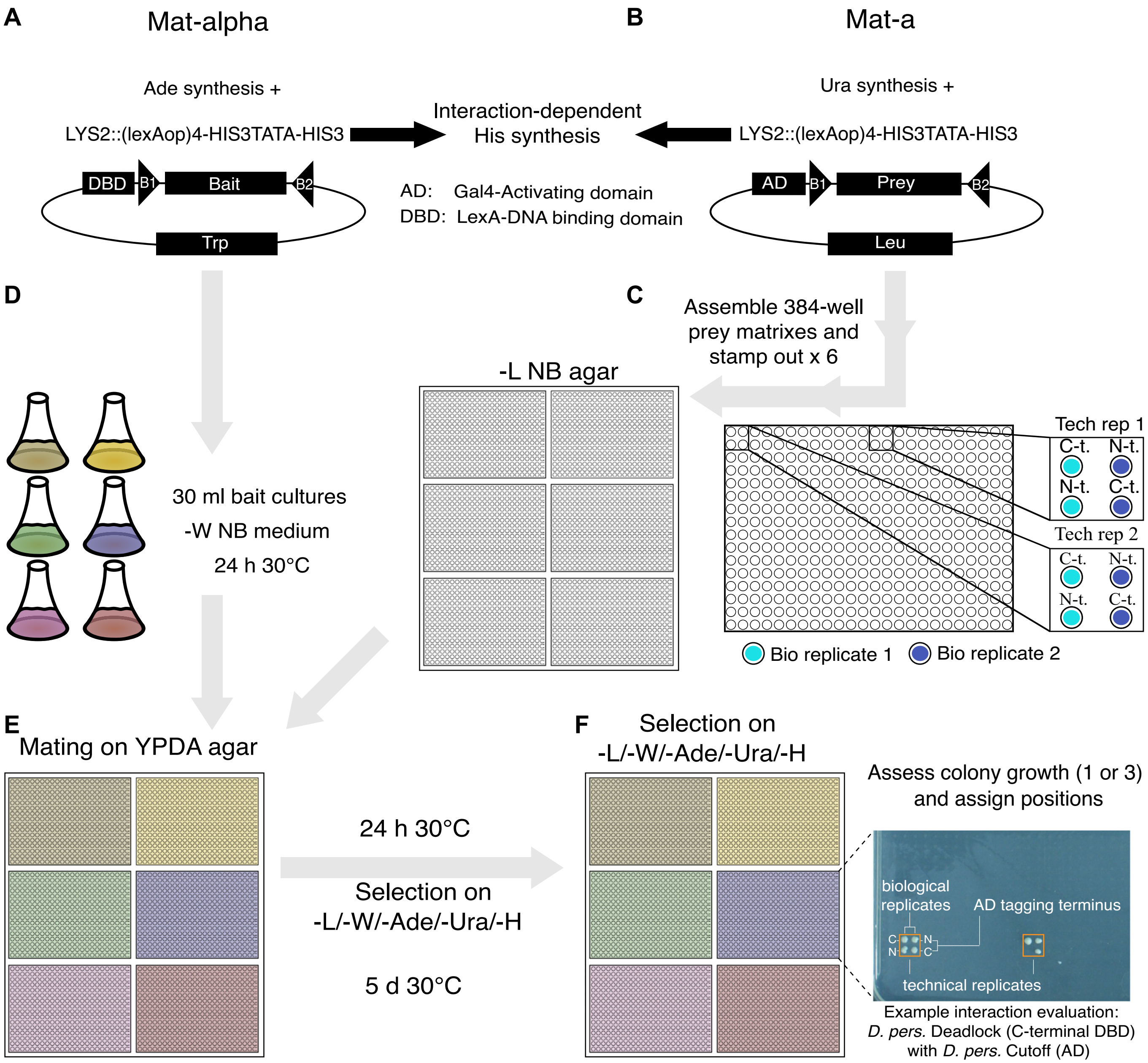

Figure EV1. Yeast-two-hybrid screen workflow.

(**A**, **B**) Mat-alpha and Mat-a strains of *S. cerevisiae* were transformed with bait (**A**) and prey (**B**) vectors, respectively, and grown in a selective medium. (**C**) Prey yeast strains were assembled into a fixed 384-well format stamped out in six replicates per tray and grown on selective medium. For mating, the prey matrices were stamped into liquid cultures of bait strains (**D**) and the mixture was transferred to non-selective YPDA medium (**E**). (**F**) After growth, the mated colonies (one bait vector per 384-format prey matrix) were stamped onto limited medium (-Leu: prey plasmid; -Trp: bait plasmid; -Ade: selection against Mat-alpha; -Ura: selection against Mat-a; -His: selection for PPI). The trays were then incubated at 30 °C for four – seven days before images were taken.

© The Author(s)

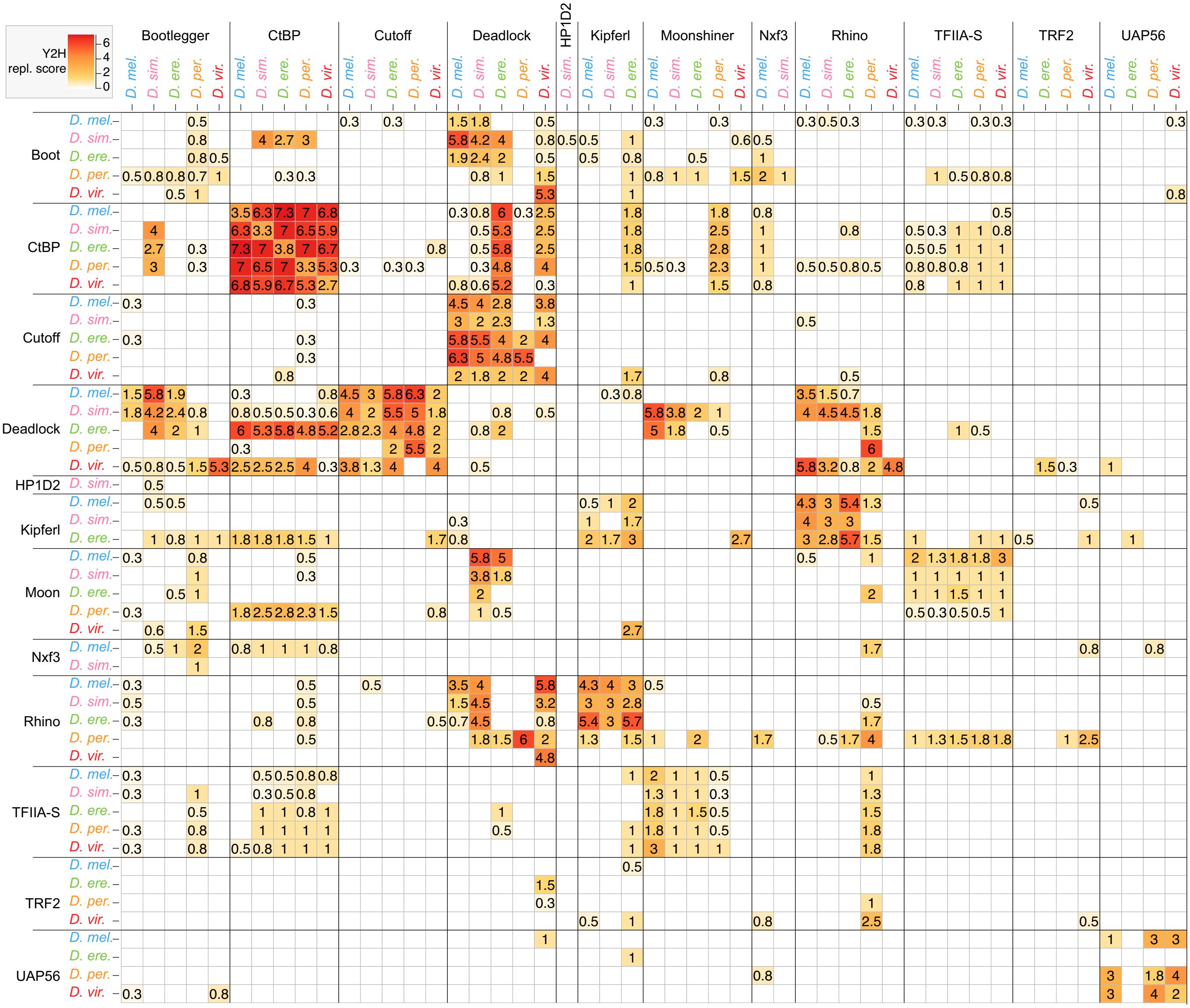

Figure EV2. Yeast-two-hybrid replication scores for all tested interactions.

Y2H replication scores are noted in the matrix as numbers and by heat map color code (see legend). The replication scores were calculated as described in Appendix Fig. S1. Of note, HP1D2 was included because it is a likely duplication of Rhino present in *D. simulans*. Since no interactions were found connecting it to the piRNA pathway, we left it out of the main text but included it here for completeness.

© The Author(s)

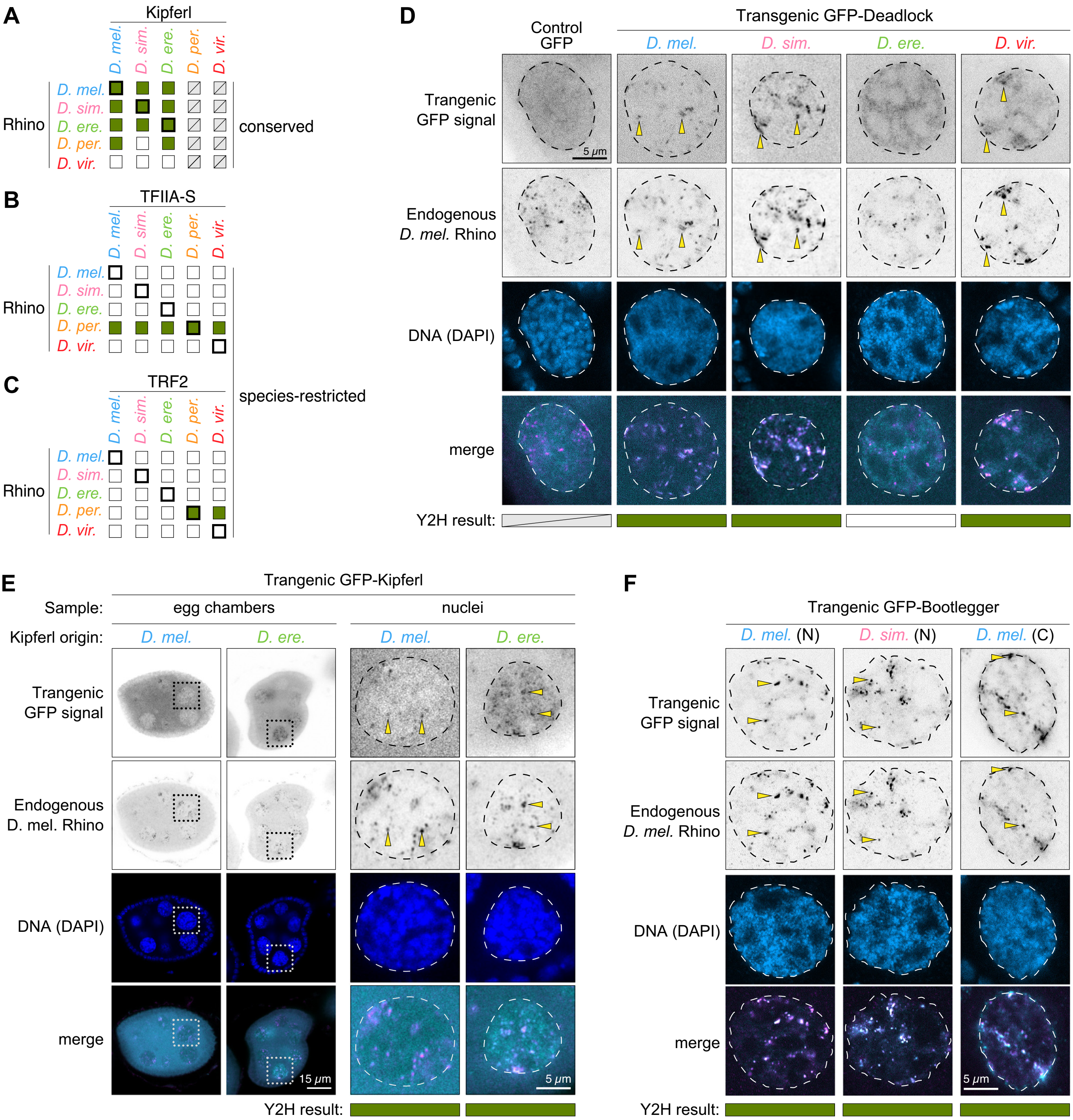

Figure EV3. Supplementary data related to Fig. 3.

(**A–C**) Summary of interactions detected by yeast-two-hybrid between orthologs of rhino and kipferl (**A**), TfIIA-S (**B**), and Trf2 (**C**). Intra-species (thick box outline) and inter-species (thin box outline) interactions are shown as filled green boxes. In contrast, empty boxes indicate the absence of interaction detection above the replication score threshold. Gray crossed-out circles for Kipferl denote the absence of the gene in those species. Pattern: evolutionary signature of the interaction (see main text for details). (**D–F**) Confocal microscopy images showing the localization of endogenous *D. melanogaster* Rhino (anti-Rhino IF) and GFP-tagged transgenic Deadlock (**D**; gray-scale images reused from Fig. 3H), Kipferl (**E**; gray-scale nuclei images reused from Fig. 3I), and Bootlegger (**F**) from the indicated species. Dashed line: nuclear border as determined by DAPI staining. Yellow arrows highlight co-localizing foci of endogenous Rhino IF signal and transgenic GFP-tagged proteins. Scale bars indicate 5 µm. Source data are available online for this figure.

 © The Author(s)

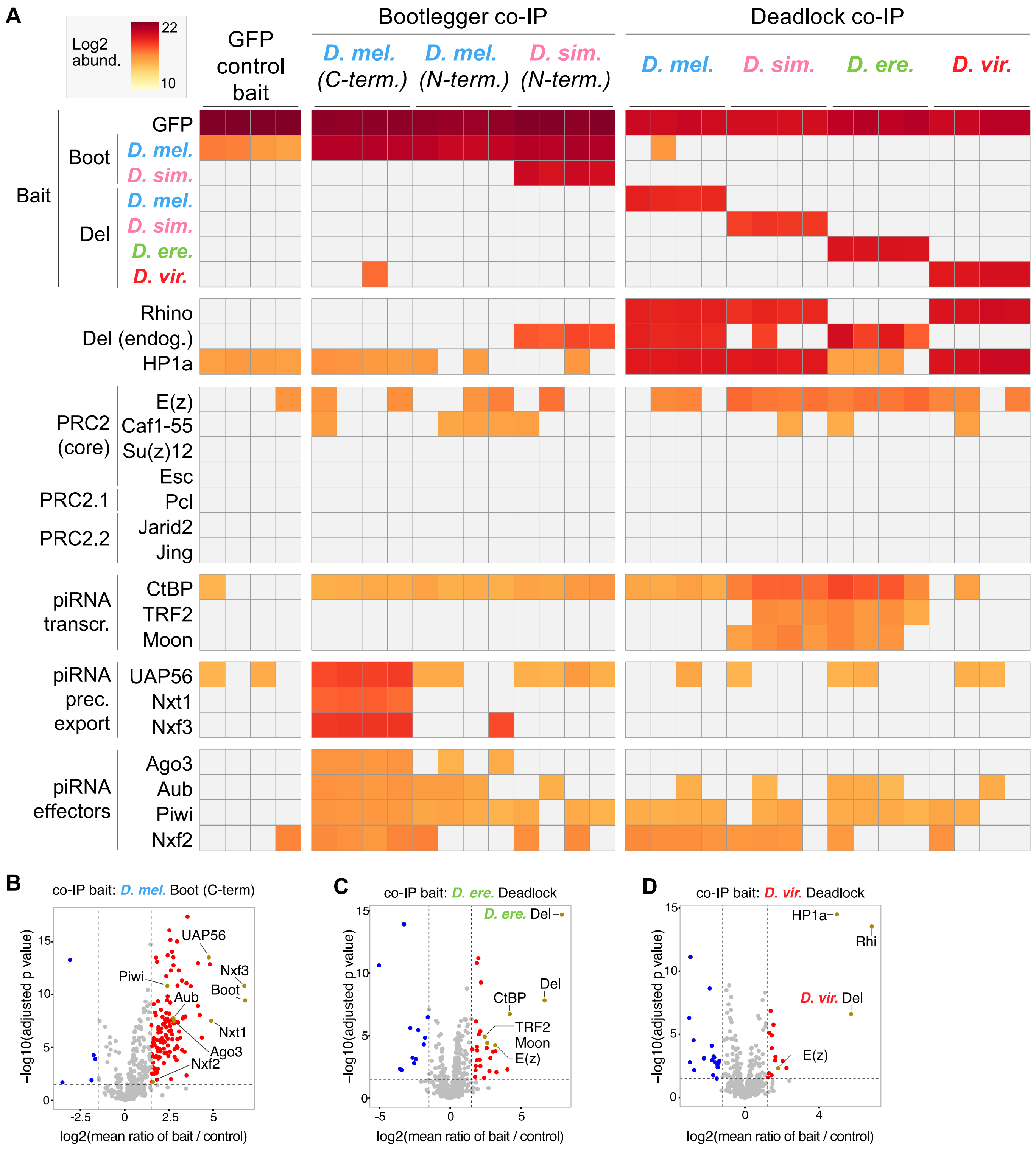

Figure EV4. Supplementary co-IP/MS data related to Fig. 4.

(A) Heatmap diagram showing log2-transformed mass spectrometry abundance signal values for the proteins indicated to the left in the co-IP/MS bait samples denoted above the diagram. (B–D) Volcano plots displaying co-IP/mass spectrometry data from the co-IP baits indicated above each plot. The x axes show log2 fold change between bait and control IP averaged over four biological replicates with each two technical replicates. The *y* axes display the negative log10 of adjusted *P* values from *t* tests for enrichment in bait compared to control co-IP. Red and blue dots represent proteins with more than 1.5-fold change with -log10(adjusted *P* values) higher than 2. Source data are available online for this figure.

© The Author(s)

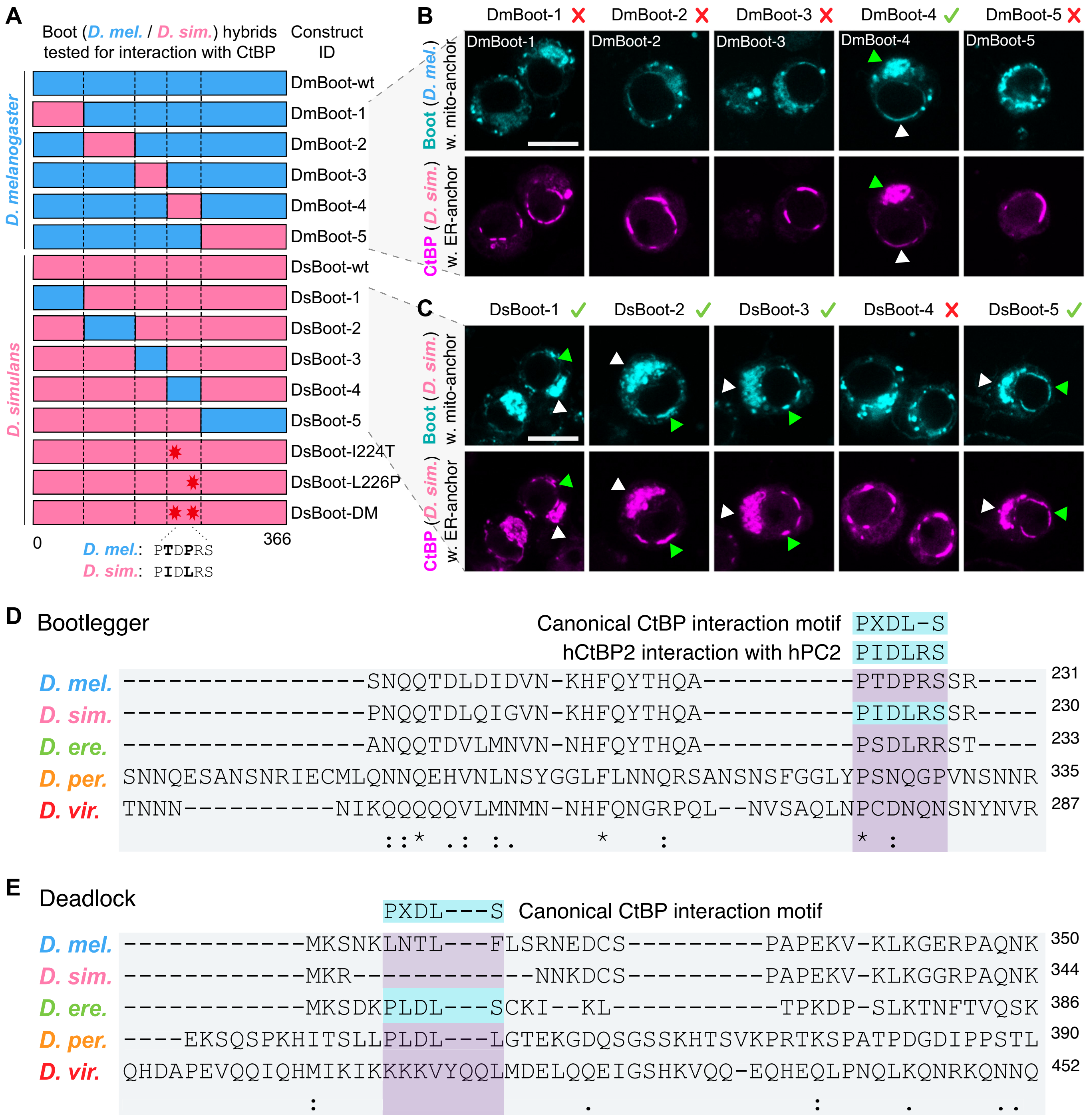

Figure EV5. Supplementary ReLo protein interaction analyses related to Fig. 5.

(**A**) Schematic representation of domain-swap constructs between the *D. melanogaster* (blue) and *D. simulans* (pink) Bootlegger orthologs with the interaction results indicated to the right by red crosses or green check marks. Black stars indicate the tested interaction site point mutants. (**B, C**) Fluorescence microscopy images of ReLo protein interaction assays testing interaction between CtBP and *D. melanogaster* Bootlegger proteins harboring swapped domains from *D. simulans* (**B**) or vice versa (**C**); see also (**A**). Shown are representative cells from >10 images. Green arrows indicate protein accumulation in interrupted ring structures around the nucleus. White arrows show protein accumulation at cytoplasmic sites. Scale bars indicate 8 μm in size. Red crosses and green check marks denote whether protein interaction was concluded or not, respectively, based on the ReLo assays. (**D, E**) Amino acid sequence alignment around the identified CtBP interaction motifs in Bootlegger (**D**) and Deadlock (**E**) from the five investigated *Drosophila* species. Source data are available online for this figure.

 © The Author(s)
