# Supplementary material for: Recurrent innovation of protein-protein interactions in the Drosophila piRNA pathway
Source: EMBO J. 2025 Apr 24;45(6):1909–32. doi: 10.1038/s44318-025-00439-8 (PMC12992792; doi:10.1038/s44318-025-00439-8)
Supplement: Supplementary file 6 — Source data Fig. 2 [file Riedelbauch_2025_EV2_Y2Hmatrix_plot.pdf › SD_Figure_2/output/Riedelbauch_2025_EV2_Y2Hmatrix_plot.pdf]

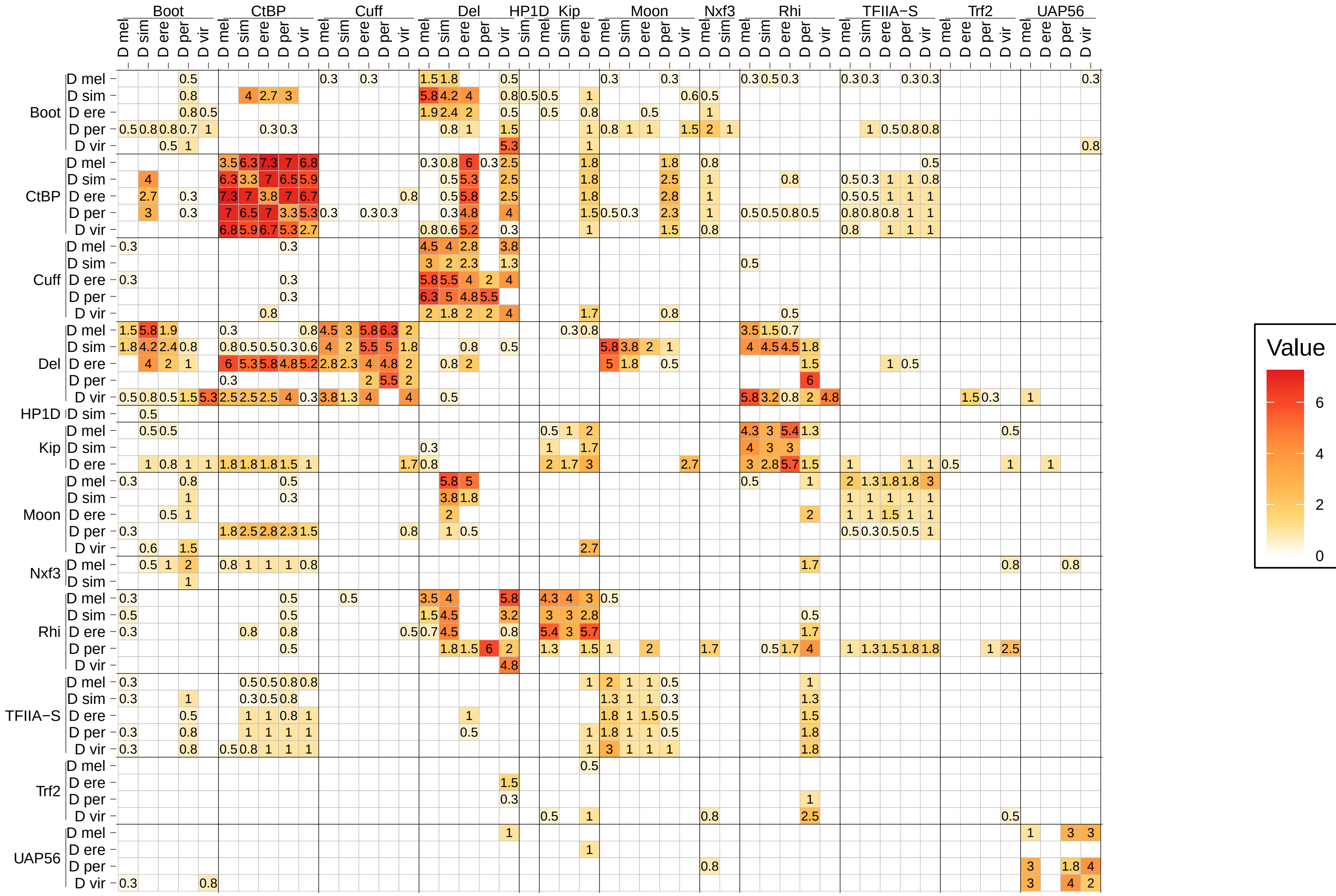

Boot
CtBP
Cuff
Del
HP1D
Kip
Moon
Nxf3
Rhi
TFIIA−S
Trf2
UAP56
D mel
D sim
D ere
D per
D vir
Value
6
4
2
0
